# Assessment of Factors Associated with Breast Self-Examination among Health Extension Workers in West Gojjam Zone, Northwest Ethiopia

**DOI:** 10.1155/2013/814395

**Published:** 2013-11-05

**Authors:** Muluken Azage, Gedefaw Abeje, Alemtsehay Mekonnen

**Affiliations:** Department of Public Health, College of Medicine and Health Sciences, Bahir Dar University, P.O. Box 79, Bahir Dar, Ethiopia

## Abstract

*Background*. Early detection of breast cancer using breast self-examination (BSE) plays an important role in decreasing its morbidity and mortality. *Objective*. To identify factors associated with BSE among health extension workers in Northwest Ethiopia. *Methods*. Cross-sectional study design was employed from October to November, 2012 in West Gojjam Zone of Amhara region. Simple random sampling technique was used to recruit a total of 390 health extension workers (HEWs). A structured Amharic questionnaire was used to collect the data. Data were entered and analyzed using SPSS statistical package version 16.0. *Result*. This study found that 37% of HEWs had ever practiced BSE and 14.4% practiced it regularly. The three main reasons for not doing regular BSE were no breast problem (53.2%), not knowing the technique of BSE (30.6%), and not knowing the importance of BSE (21.4%). Discussion with families on BSE and history of breast examination by health professionals were found significantly associated with ever practice of BSE. *Conclusion*. BSE practice was found low in this study. Having information on the importance of BSE was predictor of BSE practice. Therefore, it is important to give training on BSE techniques and its role on breast cancer prevention for HEWs.

## 1. Introduction

Globally, about 25 million people are living with cancer [1]. Recent estimates showed that cancer incidence will almost triple by 2030, with 20–26 million new cancer diagnoses and 13–17 million deaths [2]. Cancer is the second leading cause of death in the world. More than 70% of all cancer deaths occurred in low and middle-income countries [1, 3]. 

Of all types of cancers, breast cancer is the most common cancer among women both in developing and developed countries [4, 5]. It is the leading cause of death among women aged between 40 and 55 years [6]. Recent global cancer statistics indicated that breast cancer incidence is rising at a faster rate in populations of developing countries [7, 8]. Several studies reported that breast cancer is the most common cancer, and is the principal cause of cancer deaths in women and is therefore a world concern [9–17].

Early detection of breast cancer plays an important role in decreasing its morbidity and mortality. Breast self-examination (BSE) is one of the screening methods for early detection of breast cancer [18–20]. However, women in developing countries do not perform breast self-examination for various reasons [21].

Cancer was reported the second out of the ten top cancers registered at Tikur Anbesa Radiotherapy center [6]. Most healthcare facilities in Ethiopia do not have advanced laboratory investigations for diagnosing breast cancer. In resource scarce countries like Ethiopia, BSE should be promoted for early detection of breast cancer to prevent related morbidities and mortalities.

Therefore, the aim of this study was to determine BSE and identify factors associated with BSE among HEWs. Health extension workers (HEWs) are the main actors to implement health extension packages and to provide primary healthcare services at community level in Ethiopia. Thus, if BSE is integrated with these packages, deaths from breast cancer can be averted by early detection and treatment. Moreover, findings from the study can provide information on BSE for governmental health officials and other nongovernmental organizations which are working on health particularly on cancer to raise awareness amongst women about breast cancer and the role of BSE in breast cancer prevention and control.

## 2. Methods and Materials

Cross-sectional study design was employed in West Gojjam Zone of Amhara regional state, Ethiopia, from October to November, 2012. West Gojjam Zone is one of the 13 Zones of the region which had a total of 845 HEWs at the time of the study [22]. All health extension workers who were working at the time of the study in West Gojjam Zone were the study population. The total sample size was determined using single population proportion formula {*n* = [(*Zα*/2)^2^ *P*(1 − *P*)]/*d* ^2^}, where *P* = proportion of BSE among HEWs is unknown and was as assumed 50%, *Zα*/2 at 95% CI (1.96) and *d* = 5% margin of error (0.05). By considering 5% nonresponse, the final sample size was 403. HEWs who had mastectomy were excluded from the study. HEWs were selected by simple random sampling technique using list of HEWs of Zonal Health Office as sampling frame.

Data were collected using self-administered structured Amharic questionnaire. The questionnaire had three parts. The first part was about sociodemographic characteristics of respondents, part two was about knowledge of breast cancer and breast cancer risk factors, and the last part was about breast self-examination practice. One day training was given for data facilitators and three supervisors. The completeness of questionnaires was checked every day by supervisors and principal investigators. Pretest was done before the questionnaire was administered. Data were entered and analyzed using SPSS statistical package version 16.0. Descriptive statistics were used to summarize data. Logistic regression analysis was done to identify factors associated with breast self-examination. Adjusted odds ratios with 95% confidence intervals were calculated for each of independent variables using backward stepwise binary logistic regression model to control confounder effect for the outcome variable.

The study was conducted after getting ethical clearance from IRB of the Bahir Dar University. Permission was obtained from the respective Zonal and Woreda Health Offices. Informed verbal consent was obtained from the study participants after telling the objective of the study. Confidentiality and privacy were insured for collected information from the study participants.

## 3. Result

Three hundred ninety five HEWs participated in the study. The mean age of the study participants was 23.8 ± 2.7 that ranged from 16–37 years. Almost half of the study participants were married. Majority of the participants were Orthodox Christians (98%) and 91.9% had a certificate. Of the total study participants, about 29.6% and 85.1% had television and radio, respectively. Seventy two percent and 44.1% of the education status of mothers and fathers of study participants were illiterate, respectively (Table 1).

Majority of the HEWs (93.4%) had no breast problem so far and 92.3% of the participants had no history of breast cancer from their families and/or relatives. About 59% were knowledgeable on risk factors of breast cancer. Almost half of HEWs reported that every woman is at risk of acquiring breast cancer. Three hundred twenty eight HEWs reported that breast cancer is communicable and 92.7% of the participants reported that breast cancer is a killer disease. Of the total participants, 81.3% reported that breast cancer is treatable if it is detected early and only 30.4% mentioned at least one methods of screening for breast cancer (Table 2). The methods of screening for breast cancer reported by health extension workers were clinical breast examination (22.3%), breast self examination (14.4%), mammogram (3%), ultra-sound (11.1%), and X-ray (7.1%).

Of all HEWs, only 14.4% practiced BSE regularly (every month) and 147 (37.3%) HEWs reported that they practiced BSE during their life time. The three main reasons for not doing BSE were no breast problem (53.2%), not knowing BSE technique (30.6%), and not knowing the importance of BSE (21.4%). One hundred thirty (32.9%) HEWs had discussions with families on the importance of BSE and 24.3% of participants had got information on BSE from health professionals. Almost 90% of HEWs had never examined their breast by health professionals (Table 3).

In bivariate analysis, breast problem so far, discussion with families on the importance of BSE, and history of breast examination by health professional were significantly associated with practice of BSE among HEWs (Table 4). However, discussion with families on the importance of BSE and ever had breast examination by health professionals remained significantly associated with practice of BSE by entering those variables, whose *P* value is less than 0.2 in multivariate analysis. Those HEWs who had discussion with families on the importance of BSE {AOR : 5.51, 95% CI: (3.45, 8.79)} is 5.51 times more likely to practice BSE than those HEWs who had no discussion with someone on the importance of BSE. Similarly those HEWs who examined their breast by health professional {AOR : 2.69, 95% CI : (1.31, 5.52)} were 2.69 times more likely to practice BSE than their counter parts (Table 4).

## 4. Discussion

Breast self-examination (BSE) is an important and inexpensive method for early detection of breast cancer [23]. In this study, ever practice of BSE was reported by 37.3% of HEWs which is inconsistent with studies done among Ethiopian female healthcare professionals in Addis Ababa (75.1%) [24], Nigerian Nurses in Lagos general hospital (89%) [11], female health workers in a Nigerian urban city (95%) [25], Egyptian nurses (56.4%) [26], nurses in United Arab Emirates (84.4%) [27], and registered Nurses in Singapore (63%) [28]. This could be due to the level of education of study participants and the availability of mass media. In this study the availability of TV in the study group was low (30%) and only 4.3% of HEWs took training on BSE.

Of all HEWs only 14.4% performed BSE on monthly basis in this study which is inconsistent with other studies done among healthcare professionals in Addis Ababa Ethiopia (35.5%), Egypt (18.8%), and Nigeria (82%) [11, 25, 26]. This could be due to the difference of education level among the study participants.

In this study, about 30.4% of HEWs mentioned at least one methods of breast cancer screening which is incomparable to the studies done in Addis Ababa, Ethiopia (81.4%), Egypt (81%), and Nigeria (80.7%) [11, 25, 26]. Similarly only 24.3% of HEWs in this study had information about BSE which is inconsistent with the study done in Addis Ababa Ethiopia (77.6%), Egypt (69%), and Nigeria (46%) [11, 24, 25]. This difference could be due to the level of education of the study participants.

In this study, 59.0% of the study participants were knowledgeable about breast cancer risk factors. This result is incomparable with the studies done in Addis Ababa Ethiopia (85%), Egypt (73%), and Nigeria (63%) [11, 24, 25]. The difference may be due to difference in educational status, study area and accessibility to information, composition of the study population, and accessibility to mass media.

In many findings, practice of BSE was determined by the awareness of women or having of information of women about diagnostic methods of breast cancer [12, 15, 24–26, 29]. A study done in Nigeria reported that higher level of education was statistically significant associated with BSE practice [11]. This finding is consistent with the above findings that having information on BSE from health professional is a predictor of BSE practice, and those HEWs who examined their breast by health professional were also a significant predictor of BSE practice in this study which is consistent with other studies [24, 28].

In conclusion, practice of breast self-examination was low among health extension workers. Getting information on breast self-examination from health professional, discussion with families on the importance of BSE, and history of breast exam by health professional were the factors found associated with BSE practice. Therefore, training on breast self-examination should be given to increase the practice of breast self-examination which in turn would have the impact to avert the sever morbidity and mortality of breast cancer. Moreover, giving training on the techniques of breast self-examination for health extension workers is the way of reaching the wider community and the best opportunity to tackle the problem at wider perspective.

## Figures and Tables

**Table 1:** Sociodemographic characteristics of health extension workers in West Gojjam Zone, Northwest Ethiopia, October 2012.

Variables	Total study participants	
	Number (395)	Percent
Age		
15–24	266	67.3
25–34	126	31.9
35–44	3	0.8
Marital status		
Married	196	49.6
Unmarried	155	39.2
Widowed	5	1.3
Divorced	7	1.8
Union	32	8.1
Education status		
Certificate	363	91.9
Diploma	32	8.1
Religion		
Orthodox	387	98
Muslim	5	1.2
Protestant	3	0.8
TV at home		
Yes	117	29.6
No	278	70.4
Radio at home		
Yes	336	85.1
No	59	14.9
Father education status		
Illiterate	174	44.1
Read and write	181	45.8
Elementary school	22	5.6
Secondary school and above	18	4.6
Mother education status		
Illiterate	285	72.2
Read and write	95	24.1
Elementary school	8	2.0
Secondary school and above	7	1.8

**Table 2:** Knowledge of HEWs on breast cancer in West Gojjam Zone, Northwest Ethiopia, October 2012.

Variables	Total study subject	
	Number (395)	Percent
Knowledge of risk factors of breast cancer		
Knowledgeable	233	59
Less knowledgeable	162	41
Every woman has a chance of acquiring breast cancer		
Yes	191	48.1
No	204	51.6
Breast cancer is communicable		
Yes	67	17.0
No	328	83.0
Breast cancer is a killer disease		
Yes	366	92.7
No	29	7.3
Know at least one methods of screening for breast cancer		
Yes	120	30.4
No	275	69.6
If breast cancer is early detected, it is treatable		
Yes	321	81.3
No	74	18.7

**Table 3:** Breast self examination practice among HCWs in West Gojjam Zone, Northwest Ethiopia, October 2012.

Variable	Number	Percent
Ever practiced breast self-examination (*n* = 395)		
Yes	147	37.3
No	248	62.7
How frequent have you practiced BSE (*n* = 395)		
Every month	56	14.4
Once every 6 months	70	17.9
Once every 12 months	21	5.4
Never practiced	248	62.7
Reasons for not doing breast self-examination (*n* = 248)*		
I don't have breast problem	132	53.2
I don't think as I should be examined	44	17.8
It is not comfortable	5	2.0
I don't know the technique	76	30.6
Carelessness	22	8.9
I don't believe its importance	11	4.4
I don't know its importance	53	21.4
Discussion with someone on the importance of BSE (*n* = 395)		
Yes	130	32.9
No	265	67.1
Got information on BSE by health professional (*n* = 395)		
Yes	96	24.3
No	299	75.7
History of breast examination by health professional? (*n* = 395)		
Yes	43	10.9
No	352	89.1

*Multiple responses are there.

**Table 4:** Factors associated with breast self-examination among HEWs in West Gojjam Zone, Northwest Ethiopia, October 2012.

Variables	Ever practice of BSE		COR (95%CI)	AOR (95%CI)
	Yes	No		
Age				
15–24	102	164	1.24 (0.11, 13.89)	
25–34	44	82	1.16 (0.75, 1.80)	
35–44	1	2	1.00	
TV at home				
Yes	47	70	1.20 (0.40, 1.50)	
No	100	178	1.00	
Radio at home				
Yes	128	208	1.30 (0.60, 1.65)	
No	19	40	1.00	
Training on BSE				
Yes	7	10	1.19 (0.44, 3.20)	
No	140	238	1.00	
History of breast problem				
Yes	15	11	**2.45 (1.09, 5.49)**	
No	132	237	1.00	
History of breast cancer from families/relatives				
Yes	6	22	0.43 (0.17, 1.10)	
No	141	226	1.00	
Discussion with someone on the importance of BSE				
Yes	84	46	**5.86 (3.37, 9.79)**	**5.51** (**3.45, 8.79**)*
No	63	202	**1.00**	1.00
Got information on BSE from health professional				
Yes	50	46	**2.26 (1.42, 3.61)**	
No	97	202	1.00	
Ever had breast examination by health professional				
Yes	28	15	3.65 (1.88, 7.10)	2.69 (1.31, 5.52)*
No	119	233	1.00	1.00
Know at least one method of breast cancer examination				
Yes	51	69	1.38 (0.89, 2.14)	
No	96	179	1.00	

COR: crude odds ratio, AOR: adjusted odds ratio, CI: confidence interval, BSE: breast self-examination, and HEWs: health extension workers, *P < 0.05.

## List of Abbreviations

AOR: Adjusted odds ratio
BSE: Breast self-examination
CI: Confidence interval
COR: Crude odds ratio
HEW: Health extension workers
SPSS: Statistical packages for social science
WHO: World Health Organization.
